# Taxon‐dependent effects of dispersal limitation versus environmental filters on bryophyte assemblages―Multiple perspective studies in land‐bridge islands

**DOI:** 10.1002/ece3.9844

**Published:** 2023-02-24

**Authors:** Dandan Li, Feng Zhang, Guangyu Luo, Ting Zhang, Jinqiao Lv, Wenchao Wang, Jun Yang, Dejun You, Nanlong Xu, Shuiliang Guo, Jing Yu

**Affiliations:** ^1^ College of Life Sciences Shanghai Normal University Shanghai China

**Keywords:** beta diversity, bryophyte, dispersal limitation, null model, species composition

## Abstract

To explore the taxon‐dependent contribution of dispersal limitation versus environmental filters to bryophyte assemblages. We investigated bryophytes and six environmental variables on 168 islands in the Thousand Island Lake,China. We compared the observed beta diversity with the expected values based on six null models (EE, EF, FE, FF, PE, and PF), detected the partial correlation of beta diversity with geographical distances. We quantified the contributions of spatial versus environmental variables and island isolation per se to species composition (SC) using variance partitioning. We modeled the species‐area relationships (SARs) for bryophytes and the other eight biotas. To explore the taxon‐dependent effects of spatial versus environmental filters on bryophytes, 16 taxa including five categories (total bryophytes, total mosses, liverworts, acrocarpous, and pleurocarpous mosses) and 11 species‐richest families were included in the analyses. The observed beta diversity values were significantly different from the predicted values for all 16 taxa. For all five categories, the observed partial correlations between beta diversity and geographical distance after controlling environmental effects were not only positive, but also significantly different from the predicted values based on the null models. Spatial eigenvectors are more important in shaping SC than environmental variables for all 16 taxa except Brachytheciaceae and Anomodontaceae. Spatial eigenvectors contributed more to SC variation in liverworts than in mosses and in pleurocarpous mosses than in acrocarpous mosses. The effects of island isolation on SC were significant for all five categories, highly varied at the family level. The z values of the SARs for the five bryophyte categories were all larger than those of the other eight biotas. In subtropical fragmented forests, dispersal limitation exerted significant, taxon‐dependent effects on bryophyte assemblages. It was dispersal limitation rather than environmental filtering that predominantly regulated the SC patterns of bryophytes.

## Graphical abstract

In subtropical fragmented forests, dispersal limitation exerted significant, taxon‐dependent effects on bryophyte distribution. It was dispersal limitation rather than environmental filtering that predominantly regulated SC patterns for most categories of bryophytes.

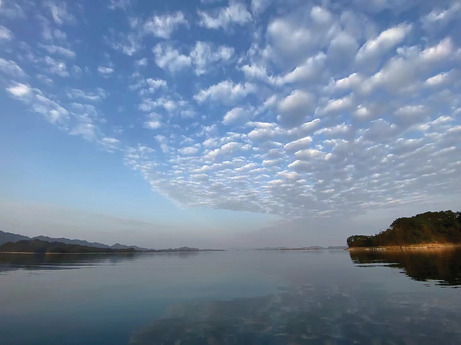

## 1 INTRODUCTION

Species assembly processes can be divided into deterministic processes (e.g., environmental filtering) based on the niche theory (Baldeck et al., 2013; Chase & Leibold, 2003) and stochastic processes (e.g., dispersal limitation) based on the neutral theory (Hubbell, 2001). The relative contribution of stochastic and deterministic processes as drivers of community assembly in fragmented landscapes remains one of the central issues in ecology (Chase, 2010; Rosindell et al., 2012). The contributions of environmental versur dispersal filtering to community assemblages may vary depending on the types of organisms involved (Liu et al., 2015; Padial et al., 2014), space scale (Giladi et al., 2014; Liu et al., 2015), and environmental context (Liu et al., 2015). However, most such studies focus on vascular plants in tropical or temperate ecosystems (Burns et al., 2010; Carvajal‐Endara et al., 2017; Keppel et al., 2010), and no studies on bryophytes in subtropical fragmented forests have been conducted so far.

The subtropical forest zone in China covers an extensive area of ca. 2.5 million km^2^, supporting a high biodiversity. Most subtropical forests in China were fragmented to varying degrees because of long intensive human disturbances and land utilizations (Liu et al., 2016). Bryophytes are an important component of subtropical forests (Cai et al., 2017; Chen et al., 2015). As the second species‐richest group of green land plants and the only haploid land plants (Frahm, 2008), bryophytes exhibit specific eco‐physiological features (He et al., 2016) and life‐history traits coupled with unique diversity and distribution patterns (Kürschner & Frey, 2012). Bryophytes usually have rapid population colonization and extinction rates, high substrate specificity, and high turnover rates of habitat patches (Pharo & Zartman, 2007; Snäll et al., 2005). Their small and light spores are primarily dispersed by wind (During & van Tooren, 1987), and many have large distribution ranges (Söderström, 1998). Bryophytes evolved desiccation tolerance and represent an alternative strategy of adaptation to terrestrial environments (Proctor, 2000). They fundamentally differ from vascular plants in their poikilohydric strategy for water and nutrients. The survival and reproduction of bryophytes highly depend on external environments (He et al., 2016). Therefore, the results from studies of tracheophytes cannot be generalized to bryophytes (He et al., 2016). We speculated that the effects of dispersal versus environmental filtering on bryophyte assemblages in fragmented habitats are likely different from those of other biotas. However, we know little about this.

Wind dispersal is the most common dispersal mechanism for bryophytes (Porley & Hodgetts, 2005). Bryophytes have a long‐distance dispersal capability by spores, even by vegetative propagules (Frahm, 2008; Medina et al., 2011), and substantially lower levels of endemic speciation (Patiño et al., 2014). In theory, the long‐distance dispersal abilities of bryophytes should weaken the effects of geographical isolation (Patiño et al., 2013, 2014). To detect the effect of dispersal limitation in community assemblages, ecologists often test the relationship of SR with isolation because isolation would reduce immigration rates and affect species richness (SR) if there existed dispersal limitation (Diamond, 1969). For example, in the beech forest islands of Lake Manapouri, New Zealand, Tangney et al. (1990) detected a weak effect of island isolation on bryophyte species richness. In the boulder habitat islands, Adirondack Mountains of New York, Kimmerer and Driscoll (2000) did not find a significant isolation effect on bryophyte SR. In a forested wetland of the Granlandet nature reserve in Sweden, isolation showed no effects on bryophyte SR (Berglund & Jonsson, 2001). Yu et al. (2019b) investigated the bryophytes on 18 continental islands of the Shengsi Archipelago in the East China Sea and found that the isolation of islands did not exert significant effects on bryophyte SR and species composition (hereafter referred to as SC). Liu et al. (2020) found no significant effect of dispersal limitation on beta diversity of bryophytes in two fragmented island systems. Additionally, Medina et al. (2011) suggested that the significantly low rates of endemism in bryophytes were attributed to their long‐distance dispersal.

However, uncertainties still exist in the contribution of dispersal limitation to bryophyte assemblages (Barbé et al., 2016). For example, having tested the spatial autocorrelation of bryophytes in 49 localities, Chen et al. (2015) found a significant spatial structure for bryophyte richness. Using a spatially explicit approach, Smith and Stark (2014) found that bryophyte diversity was the joint result of dispersal limitations and the distinctively spatially patterned environment of desert shrub lands. Based on variation at six nuclear microsatellite loci in 50 populations of *Platyhypnidium riparioides* in southern Belgium, Hutsemékers et al. (2010) detected a strong dispersal limitation at the landscape scale. Snäll et al. (2005) found that the colonization probability of *Neckera pennata* (Musci, Neckeraceae) was positively correlated with the connectivity of their host trees. In northeastern Brazil, Silva et al. (2014) demonstrated significant correlations between the distance matrix and bryophyte floristic matrix, whereas correlations between the environmental matrix and the floristic matrix were not significant. Tiselius et al. (2019) found that both habitat filtering and dispersal capacities affected the community assembly of bryophytes on land uplift islands, and asexual mosses and liverworts showed a dispersal limitation at a landscape scale of 10 km. Zartman et al. (2006) found that patch colonization rates of bryophytes declined as the intensity of fragmentation increased in a transplant experiment with two epiphyll species, which provided supporting evidence for dispersal limitation.

Based on the datasets of bryophyte communities from the Thousand Island Lake, Zhejiang, China (hereafter referred to as TIL), Liu et al. (2020) found no significant difference between observed and expected beta diversity based on the FF null model, and no significant correlation of bryophyte beta diversity with island isolation in the archipelagos. Therefore, they thought that there was no significant effect of dispersal limitation on bryophyte species assemblages in their study geographical range (Liu et al., 2020). However, the dataset used by Liu et al. (2020) was restricted to a relatively narrow geographical range, consisting of only 10 islands within a geographical range of ca. 3.2 km^2^. How about the conclusion will be if the dataset from the whole archipelago? Additionally, Liu et al. (2020) did not quantify the influences of environmental heterogeneity among islands on their conclusions, and the expected values only based on the FF null model are invulnerable to Type I error. Therefore, further studies are necessary for a better understanding of the relative contribution of dispersal limitation versus environmental filters to bryophyte assemblages.

Bryophytes highly vary in the number of spores and vegetative propagules, as well as in the frequency of producing sporophytes (Imura, 1994; Pei et al., 2010; Wills et al., 2018; Zanatta et al., 2020) which would influence their dispersal capacities (Goffinet & Shaw, 2000; Löbel & Rydin, 2010). Different taxa of bryophytes also highly vary in the responses of their SR and SC to environmental factors such as island area, elevation, shape irregularity, and vegetative coverage (Yu, Shen, Li, & Guo, 2019; Yu, Shen, Zang, Cai, & Guo, 2019). Therefore, we speculated that the relative contribution of dispersal limitation versus environmental filtering to bryophyte assemblages in subtropical fragmented forests is possibly taxon‐dependent (namely, varies among different taxa).

Land‐bridge islands formed by damming and subsequent inundation are formed at the same time as a result of a single known disturbance event. These islands have well‐delineated boundaries and are surrounded by water; they are thus an ideal system to study the contribution of dispersal limitation versus environmental filtering to bryophyte assemblages. Using multiple perspective studies, including the partial Mantel test, variance partitioning, PCNM (principal coordinates of neighbor matrices), modeling SARs, and comparison of observed beta diversity with expected values based on null models, we aimed to clarify: (1) to what extent dispersal limitation versus environmental filtering affect bryophyte assemblages in subtropical fragmented forests; and (2) whether different bryophyte taxa vary in their response to dispersal limitation versus environmental filtering.

## 2 MATERIALS AND METHODS

### 2.1 Study area

**FIGURE 1:**
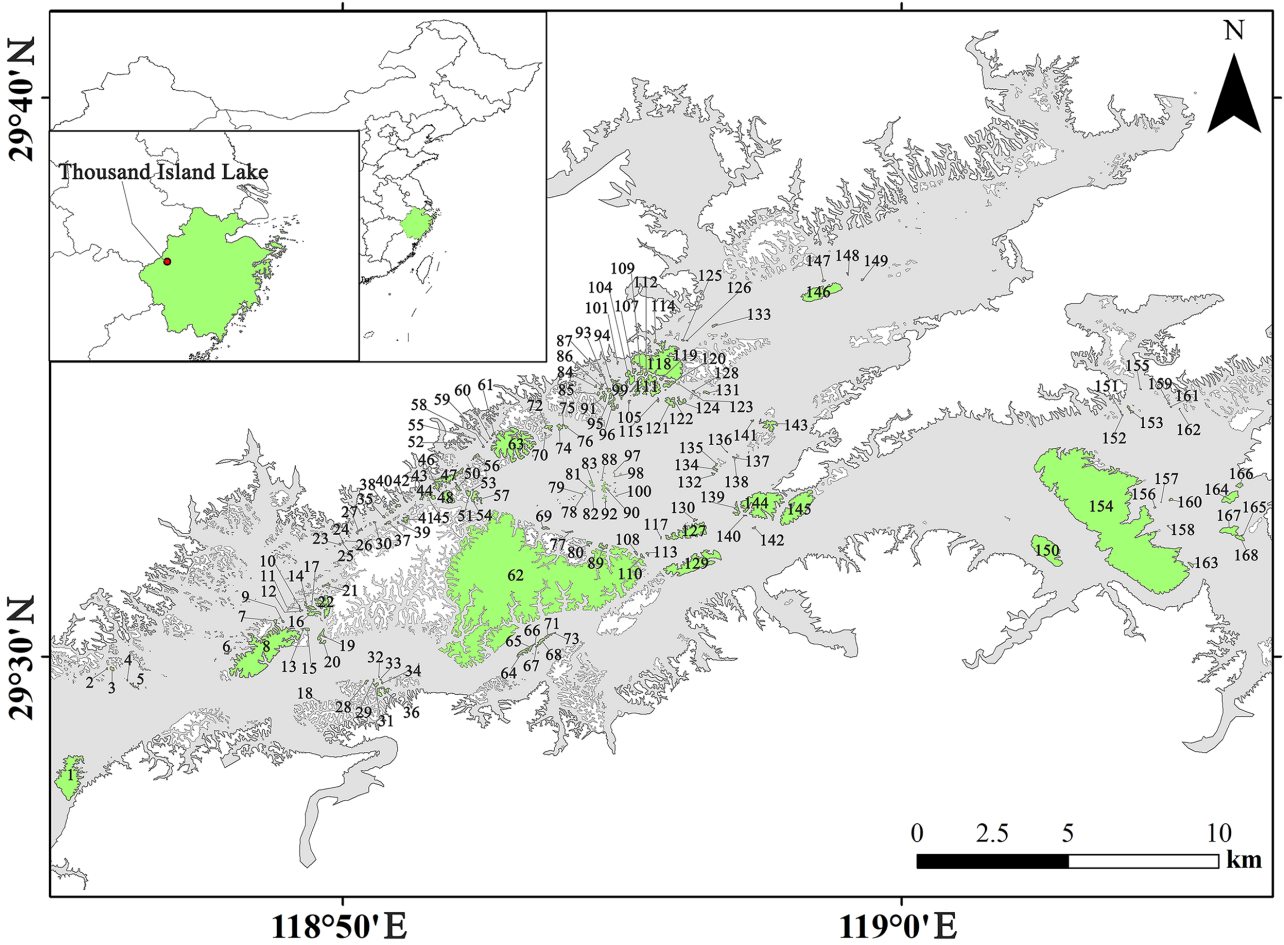
Localities of 168 islands in the Thousand Island Lake.

The Thousand Island Lake (hereafter referred to as TIL, 29°22′–29°50′ N, 118°34′–119°15′ E, Figure 1) is a large hydroelectric reservoir that was created in 1959 by the damming of the Xinanjiang River in the western Zhejiang Province, China (Wang et al., 2009). With the construction of the dam, an area of 573 km^2^ was inundated, forming 1078 islands (>0.25 ha) out of former hilltops when the water reached its final level (108 m). The elevation in TIL varies from 110 to 250 m, with a maximum of 405.2 m (Wang et al., 2018). The Thousand Island Lake is located in the middle of the subtropical zone with a typical subtropical monsoon climate, which is highly seasonal, hot in summers and cold in winters. The average annual temperature is 17.0°C, ranging from −7.6°C in January to 41.8°C in July. Annual precipitation in the region is ca. 1430 mm (Wang et al., 2018). During dam construction, primary forests in the region were clear‐cut, resulting in near complete deforestation before the lake's inundation. Currently, most islands (formerly hilltops) are covered with naturally secondary forests dominated in the canopy by *Pinus massoniana*, which are mixed with some broad‐leaved trees and shrub species in the subcanopy and understory. The forests were formed by secondary succession through regeneration after dam construction. The Thousand Island Lake is now protected as a national park, and its vegetation has not experienced significant human disturbances since 1962 (Wilson et al., 2016). The islands in TIL are located in a relatively narrow region and basically have similar climates, which eliminates the impacts of different climate conditions on the relationships of bryophyte assemblages with fragment attributes. The Thousand Island Lake is thus an ideal locality to quantify the relative contributions of spatial versus. environmental variables to bryophyte assemblages in subtropical fragmented forests.

### 2.2 Bryophyte inventory

We surveyed bryophyte flora on 168 islands in TIL. These islands vary in a wide range of area size, degree of isolation, maximum elevation, shape, and perimeter (Table S1; Figure 1). Four field collections (Aug–Sept, 2019；Oct–Nov 2019；Nov–Dec 2020; Mar 2022) were conducted. The same team visited each island and spent a relatively comparable amount of time on each inventory (Tiselius et al., 2019). For a small island (<5.0 ha), we collected bryophyte specimens basically covering the entire island. For a medium‐sized island (5.0–10.0 ha), we collected specimens in most parts of the island (mainly along mountain ridges, cols, creaks, streams, natural, and anthropogenic roads). For a large island (>10.0 ha), according to their terrain, we used a method similar to gradsect sampling (Austin & Heyligers, 1989) to survey two approximately perpendicular strips across the center of the island (covering as many landscapes as possible). These strips often have roads, fire trails, creaks, and streams accessible by pedestrians. We restricted sampling to within 50 m of roads (fire trails or streams). In large islands, besides these strips, we also continued collection and surveying if there were other roads (or streams, fire trails) accessible for pedestrians until no additional bryophyte species and no new habitat types were found, which ensured that we obtained a relatively complete species list of bryophytes for large islands and made the data comparable. When surveying, we used a method similar to floristic habitat sampling instead of plot sampling for estimating species richness by fully sampling representative landscapes (hillsides, mountain streams, relatively natural forests, secondary forests, artificial forests, forest gaps, orchards, rock cliffs, roadsides, ditches, etc.) and microhabitats (different substrates such as soil, rocks, tree trunks and bases, decayed logs, and stumps.) (Newmaster et al., 2005).

Preliminary identification to family and genus was completed in the field. All specimens were identified in the laboratory using a microscope. Voucher specimens were deposited at the bryophyte herbarium, Shanghai Normal University (SHTU). The nomenclature followed the TROPICOS database (http://www.tropicos.org, 2022).

### 2.3 Environmental variables

Environmental variables were collected at the island level, which included island area, maximum elevation, habitat richness, shape irregularity, vegetation coverage, and isolation degree.

The isolation degree of a given island was represented by the relative proportion of water within a circle (a diameter of 500 m) centered on the island (Berglund & Jonsson, 2001) (referred to as ISW) and its shortest distance to the shore (referred to as ISD).

The habitat types on each island were enumerated following the approach used by Triantis et al. (2006), using aspects of the environment known to be important to bryophyte distribution. Definitions of each habitat type are listed in Table S3. Habitat diversity was calculated as the total number of these habitat types present on each island. We did not use NDVI to estimate vegetation coverage because many islands in the study region are too small. The vegetation coverage of each island was quantitatively estimated based on the map from Google Earth using the ImageJ Ecosystem software (Schindelin et al., 2015), which was calibrated by our observations in situ. Shape irregularity is indicative of the relative amount of edge habitat on an island, which was calculated as follows (Patton, 1975):

$$\mathrm{SHA}=\frac{P}{2\sqrt{\mathrm{A\pi}}}$$

where *P* is the perimeter in meters, *A* is the area (m^2^). The higher the SHA is, the more irregular the shape, with 1 meaning that the island is circular.

### 2.4 Data analysis

#### 2.4.1 Analysis of the credibility of specimen sampling on large islands

Previous studies showed that the number of sampling points per unit area may decline with increasing island size (Gavish et al., 2011), which resulted in a sampling bias for large islands. To make sure whether our sampling on large islands was adequate, having randomized the collection sequence of the specimens, we modeled the relationships of accumulative species with accumulative specimens using the asymptotic function to obtain expected species numbers for the large islands. The sampling error (SE) of a given island was calculated by comparing the difference between the expected species number (*E*) and the observed species number (*O*) of the island $\left(\mathrm{SE}=\frac{E-O}{E}*100\%\right)$.

To clarify the effects of dispersal limitation versus environmental filtering on bryophyte assemblages, we conducted multiple perspective data analyses from following five aspects.

#### 2.4.2 Comparison of observed beta diversity with expected values based on null models

Beta diversity is the distance or dissimilarity between each sample pair in species composition (Condit et al., 2002). It can be driven by environmental heterogeneity, dispersal limitation, and the interaction between the two processes (Condit et al., 2002; Liu et al., 2016; Mori et al., 2018). To explore the mechanisms of species assembly, null models have been often used to simulate expected beta diversity where the focal ecological process does not exist (Chase et al., 2011; Liu et al., 2020; Segre et al., 2014). When examining whether dispersal limitation affects beta diversity, null models generate species level stochasticity by randomly drawing species based on their occurrence frequency in the relevant set of islands, and species could disperse randomly and colonize successfully between islands. If the observed and expected beta diversity show no significant difference, it can be interpreted as no dispersal limitation in the ecological process (Liu et al., 2020).

Beta diversity is the extent of change in community composition in relation to a gradient of environment (Whittaker, 1960). Jaccard distance coefficient could reveal the dissimilarity between each pair of patches (islands) based on the presence/absence data of species; thus, the coefficient is a measure of beta diversity (Zhong et al. 2021). The Jaccard distance coefficient was calculated as follows (Barzoki et al., 2021):

$$\mathrm{JD}=1-C/\left(A+B+C\right).$$

Where JD is Jaccard distance coefficient between island *i* and island *j*, *C* is number of species shared between island *i* and island *j*; *A* is number of species in island *i* but not in island *j*, B is number of species in island *j* but not in island *i*.

For each pair of islands, we got a beta diversity value. Therefore, for 168 islands, we got 14,028 (168*167/2) beta diversity values for the observed matrix (or one randomized matrix based on a given null model). The observed (or expected) beta diversity in the study region is the mean of these 14,028 diversity values.

The statistical significance of beta diversity could be tested against some null hypotheses. However, which combination of metrics and null models should be used in each particular circumstance is a matter of debate (Ulrich et al., 2009; Ulrich & Gotelli, 2007, 2012). Null model algorithms were used to generate randomized matrices by which expected beta diversity was calculated. Three schemes were used in generating randomized matrices: equip—all the species or islands have equal probability when randomizing; prop—randomization according to probability proportional to observed species occurrence frequency or island species richness; and fixed—randomization maintains the species occurrence frequency or island species richness exactly the same as observed. The matrices with observed occurrences of species (row) in islands (column) were randomized using the following six null models: EE (species equip, island equip), EF (species equip, island fixed), PE (species prop, island equip), PF (species prop, island fixed), FE (species fixed, island equip), and FF (species fixed, island fixed) (Gotelli, 2000).

For a given null model, 1000 values of expected beta diversity based on 1000 randomized matrices were generated, and then these expected values were compared with the observed beta diversity by using the one‐sample *t*‐test. Randomized matrices were generated by using the “taxo.null” function in the R package “NST” (Ning, 2022).

#### 2.4.3 Partial correlation of beta diversity with geographical distance after controlling for environmental variables

The partial Mantel test was used to examine the effects of geographical distance on the beta diversity of bryophytes among islands after controlling environmental heterogeneity. The Jaccard's distance coefficients of species composition (beta diversity), geographic distance (based on Cartesian coordinates), and environmental divergence (based on island area, elevation, vegetative coverage, and habitat diversity) of each island pair for bryophytes, mosses, liverworts, acrocarpous mosses, and pleurocarpous mosses were calculated. The Cartesian coordinates were converted from the longitude and latitude using the “geoXY” function in the R package “SoDa” (Gao et al., 2019). Partial correlations of the beta diversity values with geographical distances for bryophytes after controlling the environmental heterogeneity were analyzed using the “mantel. partial” function in the R package “vegan” (Oksanen, 2022b). The significant level was tested using the permutation method (permutation times = 999).

Based on 1000 randomized matrices resulting from a given null model, 1000 expected partial correlation values of beta diversity with geographical distance after controlling the environmental heterogeneity were generated. These expected values were compared with the observed partial correlation coefficient using the one‐sample *t*‐test. Randomized matrices were generated using the above six null models (Gotelli, 2000; Ning, 2022). Significant differences between the observed and expected partial correlation coefficients can further confirm the effect of geographical distance on bryophyte beta diversity.

#### 2.4.4 Relative contributions of spatial versus environmental filtering to bryophyte assemblages by using PCNM

Although the Mantel test has been widely used for investigating the relationship between community dissimilarity and geographic distance, it usually underestimates the variance partitioned by explanatory factors (Legendre & Fortin, 2010). Therefore, the partial canonical correspondence analysis (CCA) was used to further partition variation in SC into relative proportions explained by environmental variables (filtering) and those by spatial variables (dispersal limitation) (Legendre & Legendre, 2012).

Based on Cartesian coordinates, spatial eigenvectors were obtained using the PCNM (principal coordinates of neighbor matrices) with a threshold value of minimum distance. These spatial eigenvectors represent an orthogonal series of vectors describing the spatial autocorrelation of islands from broad to fine scales (Borcard et al., 2004; Legendre et al., 2009). The spatial eigenvectors were produced using the function “pcnm” in the R package “vegan.” To avoid overestimation of explained variance, we used forward selection (“ordistep” function in the R “vegan” package) to identify significant eigenvectors (Blanchet et al., 2008). Principal coordinates of neighbor matrices eigenvectors with significant positive eigenvalues (*p* < .05) were used as spatial explanatory variables. As a result, 27 spatial eigenvectors were obtained for bryophytes on 168 islands. Environmental variables included area, maximum elevation, shape irregularity, vegetation coverage, and habitat richness. The partial CCA was performed by using the “cca” function in the R package “vegan” (Oksanen, 2022a).

#### 2.4.5 Relative contributions of isolation to bryophyte assemblages using variance partitioning

The partial CCA was also performed to partition the SC variation into parts attributed to isolation variables per se and the confounded parts between the isolation variables and the other remaining variables. When analyzing, rare species were not down weighted. The partial CCA was conducted using the CANOCO for windows 5.0 (ter Braak & Šmilauer, 2012).

#### 2.4.6 Comparison of *z* values in SARs of bryophytes with those of other biotas

The *z value* in the linearized power function log *S* = log *C* + *z* × log *A* was influenced by the degree of isolation to an extent. To compare *z* values of the SARs of bryophytes with those of other biotas, with the data of large ground‐dwelling mammals (Xu et al., 2014), small mammals (Wang et al., 2010; Zhao et al., 2009), birds (Ding et al., 2013), lizards (Hu et al., 2012), spiders (Ge, 2015), ants (Zhou et al., 2019), and vascular plants (Hu et al., 2011) in TIL, we modeled the SARs of bryophytes and the above biotas with the same area unit (m^2^).

Bryophytes are an informal group consisting of liverworts and mosses, the latter consists of acrocarpous and pleurocarpous mosses. There are differences in environmental adaptation between liverworts and mosses and between acrocarpous and pleurocarpous mosses (Kürschner, 2004; Vanderpoorten & Goffinet, 2009). Mosses are generally more drought tolerant than liverworts (Hylander et al., 2005; Kürschner, 1999). Many liverworts prefer shady habitats (Vanderpoorten & Engels, 2002). Generally speaking, acrocarpous mosses, often in turfs and cushions, usually dominate in sunny, dry, and xeric habitats, whereas pleurocarpous mosses, often in mats, wefts, tail, and fan forms, frequently dominate in shady, humid and mesic to hygric sites (Kürschner, 2004). The species of different bryophyte families have different preferences for habitats and substrates. For example, the species of Pottiaceae often occur in sunny, harsh, and dry environments (Gao, 1996; Zander & Eckel, 1993), and those of Bryaceae are short‐lived annual mosses in disturbed or open sites on bare soil such as farm fields (Li, 2000). The species of Anomodontaceae and Entodontaceae are essentially recorded from rocks and tree trunks (Wu, 2002), and those of Pylaisiadelphaceae are typical epiphytes (Wu & Jia, 2004). Compared with those of Anomodontaceae, Entodontaceae, and Leucobryaceae, the species of Brachytheciaceae, Fissidentaceae, and Mniaceae often grow on more diverse microhabitats and substrates such as soils, stones, trunks, fringe wetlands of ditches and creeks (Gao, 1994, 1996; Hu & Wang, 2002; Li, 2000). To explore taxon‐dependency in the contribution of dispersal limitation versus environmental filtering to bryophyte species assemblages, five categories and 11 species‐richest families were included in the above analyses. The five categories included total bryophytes, total mosses, liverworts, acrocarpous mosses, pleurocarpous mosses, and the 11 families included Anomodontaceae, Brachytheciaceae, Bryaceae, Entodontaceae, Fissidentaceae, Hypnaceae, Leucobryaceae, Lophocoleaceae, Mniaceae, Pottiaceae, and Pylaisiadelphaceae.

## 3 RESULTS

### 3.1 General aspects of bryophyte flora and sampling credibility

A total of 209 bryophyte species were identified based on 5669 specimens collected from the 168 islands (Figure 1; Tables S1 and S2). Among these species were one species of hornwort, 28 species of liverworts (21 genera and 15 families), and 180 species of mosses (85 genera and 35 families). Brachytheciaceae (23 species, 11.06%), Pottiaceae (19, 9.13%), Bryaceae (14, 6.73%), Hypnaceae (13, 6.25%), and Entodontaceae (10, 4.81%) were the most species‐rich families. Pleurocarpous mosses (98 species, 46.89%) were dominant over acrocarpous mosses (78 species, 37.32%).

After randomization of the collection sequence of the specimens, we found that the accumulative species increased with accumulative specimens for the eight largest islands, well following the asymptotic model (Figure S2 and Table S5). The expected total species numbers were similar to those actually observed, with larger errors for Islands 129 and 146 (10.66%), indicating that sampling on larger islands was overall adequate.

### 3.2 Beta diversity based on observed and randomized data

The observed beta diversity values for total bryophytes, total mosses, liverworts, acrocarpous mosses, and pleurocarpous mosses were 0.8629, 0.8582, 0.7928, 0.8791, and 0.8063, respectively. The observed beta diversity value was significantly higher in mosses than in liverworts (*p* < .001) and in acrocarpous mosses than in pleurocarpous mosses (*p* < .001) (Table 1). For all the 11 major families, the observed beta diversity values highly varied from 0.3274 in Mniaceae to 0.8698 in Brachythecieae (Table 2), being significantly different among them (*p* < .001) except those between Bryaceae and Entodontaceae (*p* = .715), between Bryaceae and Fissidentaceae (*p* = .188), between Entodontaceae and Fissidentaceae (*p* = .250), and between Pylaisiadelphaceae and Lophocoleaceae (*p* = .519) (Table S4). The observed beta diversity values showed that the distribution patterns were different between mosses and liverworts, and between acrocarpous mosses and pleurocarpous mosses (Table 1), and highly varied among the 11 major families (Table 2).

**TABLE 1:** Observed beta diversity and expected beta diversity based on six null models for five categories of bryophytes (df = 9999).

Categories	Observed values	Expected values based on null models					
		EE	EF	PE	PF	FE	FF
Bryophytes	0.8629	0.9479^a^	0.9617^a^	0.8675^a^	0.9093^a^	0.8496^a^	0.8628^a^
Mosses	0.85815	0.9445^a^	0.9586^a^	0.8636^a^	0.9053^a^	0.8453^a^	0.85821^a^
Liverworts	0.7928	0.9475^a^	0.9526^a^	0.8385^a^	0.8665^a^	0.8093^a^	0.7930^a^
Acrocarpous mosses	0.8791	0.9442^a^	0.9583^a^	0.8782^a^	0.9134^a^	0.8670^a^	0.8793^a^
Pleurocarpous mosses	0.8063	0.9387^a^	0.9510^a^	0.8359^a^	0.8790^a^	0.8045^a^	0.8064^a^

*Note*: EE, EF, PE, PF, FE, and FF are six null models.

^a^ Significant levels (differences from the observed values) at *p* < .001, respectively.

**TABLE 2:** Observed beta diversity and expected beta diversity based on six null models for 11 major families of bryophytes (df = 9999).

Families	Observed values	Expected values based on null models					
		EE	EF	PE	PF	FE	FF
Brachytheciaceae	0.8698	0.9384^a^	0.9484^a^	0.8853^a^	0.9099^a^	0.8845^a^	0.8701^a^
Bryaceae	0.6858	NA	0.9147^a^	0.7769^a^	0.8021^a^	0.7281^a^	0.6886^a^
Pottiaceae	0.8292	0.9189^a^	0.9250^a^	0.8496^a^	0.8728^a^	0.8367^a^	0.8304^a^
Leucobryaceae	0.6572	NA	0.8154^a^	0.6873^a^	0.7049^a^	0.6562^a^	0.6567^a^
Lophocoleaceae	0.5409	0.7910^a^	0.7829^a^	0.6263^a^	0.6581^a^	0.5628^a^	0.5380^a^
Fissidentaceae	0.7545	0.8588^a^	0.8635^a^	0.7754^a^	0.7937^a^	0.7611^a^	0.7549^a^
Hypnaceae	0.6792	0.8493^a^	0.8584^a^	0.7538^a^	0.7862^a^	0.6866^a^	0.6791^a^
Entodontaceae	0.8629	0.8722^a^	0.8785^a^	0.7597^a^	0.7914^a^	0.7058^a^	0.6844^a^
Pylaisiadelphaceae	0.5313	NA	0.7070^a^	0.5721^a^	0.5721^a^	0.5333^a^	0.5307^a^
Anomodontaceae	0.4945	NA	0.8168^a^	0.5789^a^	0.6361^a^	0.5098^a^	0.4899^a^
Mniaceae	0.3274	NA	0.8511^a^	0.5152^a^	0.5811^a^	0.3830^a^	0.3250^a^

*Note*: The meaning of the acronyms and marks are the same as those in Table 1. NA: no valid values were gotten because of no species in a pair of islands in the simulation using the EE null model.

^a^ Significant levels (differences from the observed values) at *p* < .001.

The observed beta diversity values were significantly different (*p* < .001) from the predicted values based on the six null models for all the five bryophyte categories (Table 1) and all the 11 families except those based on the EE null model because of empty columns in the randomized matrices (Table 2), which indicated nonrandom distribution patterns of species across forest fragments for the five categories and 11 major families.

### 3.3 Effect of geographical distance on beta diversity after controlling for environmental variables

The observed partial correlation coefficients of beta diversity with geographical distance (coordination) after controlling environmental heterogeneity were significant for total bryophytes (*r* = .1493, *p* < .001), total mosses (*r* = .1432, *p* < .001), liverworts (*r* = .090, *p* < .02), pleurocarpous mosses (*r* = .1257, *p* < .005), and marginally significant for acrocarpous mosses (*r* = .0471, *p* = .072) (Table 3).

**TABLE 3:** Observed partial correlation coefficients of beta diversity with geographical distance after controlling environmental heterogeneity and expected values based on six null models for bryophytes in the Thousand Island Lake (df = 9999).

Categories	Observed values	Expected values					
		EE	EF	PE	PF	FE	FF
Bryophytes	0.1493	0.0009^a^	0.0777^a^	0.0055^a^	0.1034^a^	−0.0063^a^	0.1491^b^
Mosses	0.1432	−0.0013^a^	0.0732^a^	0.0018^a^	0.1016^a^	−0.0057^a^	0.1430^b^
Liverworts	0.0901	−0.0002^a^	0.0007^a^	0.0043^a^	0.0053^a^	−0.0024^a^	0.0822^a^
Acrocarpous mosses	0.0471	0.0016^a^	0.0253^a^	−0.0024^a^	0.0389^a^	0.0078^a^	0.0467^b^
Pleurocaropus mosses	0.1257	0.0001^a^	0.0460^a^	0.0028^a^	0.0706^a^	−0.0002^a^	0.1250^b^

*Note*: The meaning of the acronyms and marks are the same as those in Table 1.

^a^ Significant levels (differences from the observed values) at *p* < .001.

^b^ Significant levels (differences from the observed values) at *p* < .5.

The observed partial correlation coefficients of beta diversity with geographical distance were significantly different (most at *p* < .001) from the predicted values based on the six null models for all five bryophyte categories (Table 3). The above results further indicated that the beta diversity of the bryophytes in the study region was affected by geographical distance (dispersal limitation).

### 3.4 Relative contributions of spatial versus environmental filtering to bryophyte SC

Spatial eigenvectors exerted stronger effects on SC than environmental variables for all five bryophyte categories and the 11 families except Brachytheciaceae and Anomodontaceae. For total bryophytes, spatial eigenvectors versus environmental variables independently explained 20.51% and 6.87% of the total SC variations, respectively. The above results indicated that dispersal limitation exerted more important effects than environmental filtering on SC, not only for total bryophytes but also for mosses and liverworts, for acrocarpous and pleurocarpous mosses, and for most major families.

Spatial eigenvectors contributed more to SC variation in liverworts (19.53%) than in mosses (17.16%), and in pleurocarpous mosses (20.78%) than in acrocarpous mosses (18.81%). The relative contributions of spatial eigenvectors versus environmental variables to SC highly varied among the major 11 families, being highest in Lophocoleaceae (43.4% vs. 21.0%), following by Mniaceae (29.5% vs. 16.4%), Hypnaceae (21.2% vs. 6.1%), Bryaceae (18.83% vs. 14.5%), Entodontaceae (15.9% vs. 9.8%), Pylaisiadelphaceae (14.2% vs. 13.1%), Pottiaceae (13.83% vs. 5.57%), Leucobryaceae (12.0% vs. 6.2%), Fissidentance (11.6% vs. 9.6%), Brachytheciaceae (14.01% vs. 15.09%), and Anomodontaceae (6.9% vs. 19.2%) (Table 4).

**TABLE 4:** Variance partitioning revealing relative effects of spatial eigenvectors versus environmental variables on SC of 16 bryophyte taxa in the Thousand Island Lake.

Taxa	Spatial effect %	Environmental effect %	Confounded effect %	Total explained effect %
Bryophytes	20.51 (df = 27, *p* < .01)	6.87 (df = 7, *p* < .01)	4.58	31.95
Mosses	17.16 (df = 23, *p* < .01)	6.74 (df = 7, *p* < .01)	4.40	28.30
Liverworts	19.53 (df = 12, *p* < .01)	10.53 (df = 7, *p* < .01)	6.12	36.18
Acrocarpous mosses	18.81 (df = 22, *p* < .01)	7.01 (df = 7, *p* < .01)	0.19	28.15
Pleurocarpous mosses	20.78 (df = 25, *p* < .01)	7.18 (df = 7, *p* < .01)	6.56	34.52
Brachytheciaceae	14.01 (df = 5, *p* < .05)	15.19 (df = 7, *p* < .1)	5.51	34.71
Pottiaceae	13.83 (df = 8, *p* < .01)	5.57 (df = 7, *p* < .05)	1.33	20.73
Bryaceae	18.83 (df = 10, *p* < .01)	14.50 (df = 7, *p* < .01)	5.72	39.05
Leucobryaceae	12.0 (df = 5, *p* < .01)	6.2 (df = 7, *p* < .01)	0.30	18.60
Lophocoleaceae	43.4 (df = 5, *p* < .01)	21.0 (df = 7, *p* < 0.01)	0.00	62.00
Fissidentaceae	11.6 (df = 5, *p* < .01)	9.6 (df = 7, *p* < .1)	2.70	23.80
Hypnaceae	21.2 (df = 14, *p* < .01)	6.1 (df = 7, *p* < .01)	1.40	28.80
Entodontaceae	15.9 (df = 11, *p* < .01)	9.8 (df = 7, *p* < .01)	3.80	29.60
Pylaisiadelphaceae	14.2 (df = 1, *p* < .01)	13.1 (df = 7, *p* < .1)	0.00	19.70
Anomodontaceae	6.9 (df = 2, *p* < .01)	19.2 (df = 7, *p* < .02)	0.90	27.10
Mniaceae	29.5 (df = 11, *p* < .01)	16.4 (df = 7, *p* < .01)	12.8	58.70

### 3.5 Effects of island isolation on bryophyte SC

Island isolation (including ISW and ISD) exerted significant effects on SC for the five bryophyte categories (most at *p* < .01). For bryophytes as a whole, isolation explained 3.9% of the total SC variation (1.8% by isolation per se at *p* < .01 and 2.1% confounded with the remaining variables), which was not too low considering the explained variation (11.4% of the total) (Table 5).

**TABLE 5:** Variance partitioning revealing isolation effects on SC of 16 bryophyte taxa in the Thousand Island Lake.

Taxa	Independently isolation effect % (df = 2)	Confounded effect %	Total explained effect (df = 7)
Bryophytes	1.8 (*p* < .01)	2.1	11.4 (*p* < .01)
Mosses	1.7 (*p* < .01)	2.1	11.1 (*p* < .01)
Liverworts	3.5 (*p* < .01)	2.3	16.7 (*p* < .01)
Acrocarpous mosses	2.0 (*p* < .01)	0.9	9.3 (*p* < .01)
Pleurocarpous mosses	1.6 (*p* < .02)	3.7	13.7 (*p* < .01)
Brachytheciaceae	4.4 (*p* < .3)	3.1	20.7 (*p* < .02)
Pottiaceae	1.8 (*p* < .2)	0.3	6.9 (*p* < .1)
Bryaceae	4.7 (*p* < .01)	0.0	20.2 (*p* < .01)
Leucobryaceae	0.6 (*p* < 1.0)	1.3	6.6 (*p* < .4)
Lophocoleaceae	5.6 (*p* < .4)	0.8	18.6 (*p* < .5)
Fissidentaceae	3.9 (*p* < .03)	0.6	12.3 (*p* < .04)
Hypnaceae	1.9 (*p* < .2)	0.3	7.5 (*p* < .02)
Entodontaceae	2.9 (*p* < .03)	0.2	13.7 (*p* < .01)
Pylaisiadelphaceae	1.3 (*p* < .7)	0.3	5.5 (*p* < .7)
Anomodontaceae	1.5 (*p* < .8)	1.8	20.1 (*p* < .02)
Mniaceae	5.8 (*p* < .03)	1.9	29.2 (*p* < .01)

*Note*: Isolation included ISW and ISD.

The effects of island isolation on SC highly varied at the family level, being significant for Bryaceae, Fissidentaceae, Entodontaceae, and Mniaceae (*p* < .05), weak for Hypnaceae and Pottiaceae (*p* < .2), and insignificant for Brachytheciaceae, Anomodontaceae, Lophocoleaceae, Leucobryaceae, and Pylaisiadelphaceae (*p* > .2) (Table 5).

### 3.6 SARs of bryophytes and other biotas

When the area was the only constraining variable, the SARs of the five bryophyte categories are listed in Table 6. Their SRs all increased linearly and significantly with increasing area, according to the ln‐transformed functions (*p* < .001). In TIL, the z values of the SAR for total bryophytes (0.3565), total mosses (0.3449), liverworts (0.2574), acrocarpous mosses (0.2958), and pleurocarpous mosses (0.3487) were all larger than those for ground‐dwelling mammals (0.2078), mammals (0.2174), small mammals (0.1096), birds (0.1377), lizards (0.1063), spiders (0.2146), ants (0.1790), and vascular plants (0.1340) (Table 6).

**TABLE 6:** Parameters for SARs of different biotas in the Thousand Island Lake.

Biotas	C	z	*r*	Reference
Total bryophytes	2.7855	0.3565	.734	–
Liverworts	0.8021	0.2574	.685	–
Total mosses	2.7196	0.3449	.734	–
Acrocarpous mosses	1.9989	0.2958	.688	–
Pleurocarpous mosses	2.1446	0.3487	.700	–
Ants	3.1965	0.1790	.892	Zhou et al. 2019
Birds	3.1707	0.1377	.819	Ding et al. 2013
Large ground‐dwelling mammals	0.7810	0.2078	.677	Xu et al. 2014
Lizards	4.0412	0.1063	.55	Hu et al. 2012
Mammals	0.8513	0.2174	.730	Xu et al. 2014
Small mammals	1.1031	0.1096	.492	Zhao et al., 2009; Wang et al. 2010
Spiders	2.2235	0.2146	.678	Ge, 2015
Vascular plants	3.9726	0.1340	.580	Hu et al. 2011

*Note*: SAR, ln (species number + 1) = *C* + *z* × ln (area, m^2^); The SARs were produced by area as the only constraining variable.

## 4 DISCUSSION

### 4.1 About the effects of dispersal limitation on bryophyte assemblages

Most bryophytes reproduce via a huge amount of small spores (Frahm, 2008) and a large variety of vegetative ways such as rhizoidal gemmae, axillary gemmae, brood bodies, detaching leaves or buds and leaf fragments (Imura, 1994). Bryophytes may therefore disperse well and over long distances by wind (Frahm, 2008; Porley & Hodgetts, 2005) and are typically regarded as extremely efficient dispersers (Vanderpoorten et al., 2019). Some previous studies even suggested that long‐distance dispersal of spores might be more common than previously appreciated (McDaniel & Shaw, 2003; Skotnicki et al., 2001) and dispersal limitation was not considered the major ecological process driving bryophyte assemblages (Liu et al., 2020; Patiño et al., 2013, 2014).

The long dispersal capacities by spores should cause bryophytes to have large ranges; however, only a few species are actually ubiquitous (Frahm, 2008). Although some bryophytes are widely distributed with weak intercontinental genetic differentiation (Mcdaniel & Shaw, 2003; Vanderpoorten et al., 2008), intercontinental disjunctive distribution in bryophytes is not necessarily related to long‐distance dispersal (Medina et al., 2011). Many bryophytes are indeed able to reproduce prolific spores and various vegetative propagules (Frahm, 2008). Mounting evidence, however, showed that dispersal limitation actually occurred in many bryophytes at different spatial scales, among different mountains or reserves (Chen et al., 2015), on continental islands (Li et al., 2022a), treefall gaps (Kimmerer, 2005), phorophytes (Hedenäs et al., 2003; Löbel et al., 2009; Löbel & Rydin, 2009), rocky outcrops (Silva et al., 2014), and desert shrublands (Smith & Stark, 2014). After conducting multiple perspective studies, we detected strong and significant effects of dispersal limitation on bryophyte assemblages in subtropical fragmented forests, further confirming the effects of dispersal limitation on bryophyte assemblages. Additionally, we found that the effects of dispersal limitation on bryophyte assemblages varied among different bryophyte taxa, namely were taxon‐dependent.

### 4.2 About the mechanisms of dispersal limitation on bryophyte assemblages

Significant effects of dispersal limitation on bryophyte assemblages were possible due to the following reasons: First, many factors exert negative effects on bryophyte long‐distance dispersal, and many bryophytes in fact have limited dispersal capabilities (Hedenäs et al., 2003). Long‐distance dispersal requires spores to reach higher altitudes in the atmosphere. Therefore, spores must be able to get into these altitudes, which is difficult for some species from the forest floor (Frahm, 2008). Another unknown factor to consider is how easily diaspores (including various vegetative propagules) can access dispersal agents (wind, running water, insect, and so on), which might be relevant for the isolation in small organisms that are found in specific microenvironments. Local habitat characteristics, such as microsite limitation (i.e., number of logs, quality of substrate, Hylander, 2009) and physical barriers limiting wind availability (i.e., canopy or stand closure, Fenton & Bergeron, 2006; Sundberg, 2013), may influence dispersal and colonization. Some bryophyte species occupy sheltered microhabitats where wind transport is very unlikely. For example, in the Southern Appalachian Mountains (USA), there are some deep, narrow gorges harboring a remarkably isolated bryophyte flora with several endemic mosses (Billings & Anderson, 1966). Therefore, bryophyte specialists often have a limited dispersal capacity (Kuussaari et al., 2009; Pimm et al., 2014).

Second, experimental studies showed that most spores of bryophytes deposited within centimeters of the parent sporophytes (During & van Tooren, 1987; Miles & Longton, 1992; Söderström & Jonsson, 1989; Wyatt, 1982). The spores of many bryophytes typically fall within the first tens of meters (Aranda et al., 2014). Spore‐trapping experiments showed that spore density quickly decreased with distance from the source, but beyond a certain threshold of distance, spore density was distance‐independent (Lönnell et al., 2012). Such a “fat‐tailed” dispersal kernel could partly explain the wide distribution of some bryophyte species and the effects of dispersal limitation on the SC of bryophytes at the regional scale in the study region.

Third, at a given spatial scale, the differential capacities of species to produce and transport sufficient numbers of propagules from source populations to given islands result in a dispersal filter. In bryophytes, there is a trade‐off concerning the production of a few, large spores or of many, small spores that controls establishment rate versus dispersal ability (Goffinet & Shaw, 2000; Löbel & Rydin, 2010). Bryophytes are also able to disperse by means of fragmentation and by specialized asexual diaspores. Fragments and asexual diaspores have an advantage in the establishment phase and are generally thought to contribute to short‐distance dispersal. Bryophytes highly vary in number and sizes of these asexual diaspores and fragments among different taxa or at the family level (Imura, 1994; Pei et al., 2010), which likely caused the differences in their dispersal capacities and in the response of their SC to dispersal limitation at a narrower region scale.

Fourth, for bryophytes, long‐distance dispersal may occur only occasionally because spore production seems to be highly constrained due to unsuccessful sexual reproduction. Two‐thirds of the mosses are dioecious. Fertilization is only possible if male plants grow near female plants because spermatozoids can swim only a short distance, and raindrops with spermatozoids can hardly reach more than 1 m. Due to this, the chance of producing spores is quite low. Therefore, only part of the bryophytes produce spores, and many are only known in sterile conditions (Frahm, 2008). Most bryophytes reproduce mainly by asexual propagation (Miles & Longton, 1992). However, with increasing mass of the asexual propagule, the probability of transport by air streams decreases, resulting in dispersal over only short distances, especially in close forests (Pohjamo et al., 2006).

Additionally, there are indeed intercontinental distribution for many bryophytes (Frahm & Vitt, 1993), but such wide distribution was not necessarily related to long‐distance dispersal (Medina et al., 2011), may be partially attributed to an old common stock of Pangaean (or Laurasian and Gondwanan) taxa and later continental drift (Frahm & Vitt, 1993), or to step‐by‐step (stone‐stepping) dispersal over time (Patiño et al., 2015).

### 4.3 About the taxon‐dependency of the dispersal limitation effects

Effects of dispersal limitation on species distribution were stronger for liverworts than for mosses, which was consistent with the study of the bryophytes in the continental islands of the Zhoushan Archipelago (Li, Guo, et al., 2022). The difference in dispersal limitation between liverworts and mosses was attributed to the difference in their dispersal capacity. During and van Tooren (1987) suggested that the dispersal ability in mosses is stronger than in liverworts because mosses often produce more spores per sporophyte and have a higher proportion of species bearing sporophytes than liverworts.

At family level, the spatial effects on Pottiaceae and Anomodontaceae were 13.83% and 6.9%, respectively, which were relatively weaker than those of most other families. According to our field experience, Pottiaceae has more sporophyte‐bearing species than other families such as Mniaceae, Hypnaceae, and Lophocoleaceae. Additionally, for many species of Pottiaceae, specialized asexual reproduction is rather common, either by multicellular gemmae borne on stalks in the leaf axils or on leaves, or by obovoid brood bodies borne on rhizoids in the soil (Zander & Eckel, 1993). Many species of Anomodontaceae are easily dispersed by their leaf fragments. Therefore, Anomodontaceae and Pottiaceae were expected to have a stronger dispersal capacity than other families, and thus dispersal limitation exerts relatively weak effects on them.

Mosses highly vary in their sporophytes and peristome teeth, with five types of spore dispersal including decay dispersal, wind dispersal, vapor‐wind dispersal, water dispersal, and insect dispersal (Gao et al., 2000). The dispersal capacity is different for mosses with different types of spore dispersal because some dispersal agents are easily reached by moss spores, while others are not, such as insect dispersal spores in Splachnaceae (Gao et al., 2000). Spore release is affected by tooth structures. For example, the tooth structures of the genus *Diphyscium*, *Buxbaumia* do not have an active function in their spore dispersal, while the capsules of the vapor‐wind dispersal subtype have well‐developed opercula, annuli, and peristome teeth. Around 80% of the mosses belong to vapor‐wind dispersal subtypes such as *Acrocarpi‐Haplolepideae*, *Acrocarpi‐Diplolepideae*, and *Pleurocarpi‐Diplolepideae*, including Funariaceae, Bryaceae, and Orthotrichaceae (Gao et al., 2000; Medina et al., 2011). The peristome teeth of the vapor‐wind subtype are very sensitive to humidity and active in the spore dispersal process (Gao et al., 2000). For some species of Sphaerocarpales and Marchantiales, the dehiscence of the capsule takes place with the decaying of the capsule wall, and their spores are large and few in number, which limits their dispersal over a long distance (Nath & Asthana, 2001). The size of spores varies among different bryophytes (Li et al., 2022b), while the size of spores is closely relevant to the dispersal capacity. In general, small spores are correlated with long‐distance dispersal, whereas large spores have strong tendencies for short‐distance and step‐by‐step dispersal (Kürschner & Frey, 2012).

### 4.4 About the *z* values of SARs and their implication for dispersal limitation

In TIL, the *z* values of SARs were different between liverworts and mosses, and between acrocarpous mosses and pleurocarpous mosses, which was possibly related to their different sensitivities to island habitats, and to their different dispersal capacities. The *z* values in the SARs were affected by the degree of isolation in fragmented island systems (Rosenzweig, 1995), and high *z* values in the SARs indicated substantial isolation and a low immigration rate of biotas among islands (MacArthur & Wilson, 1967). Previous studies reported the z varied from 0.20 to 0.40 for true islands (Connor & Mccoy, 1979; MacArthur & Wilson, 1967; Matthews et al., 2015; Rosenzweig, 1995; Triantis et al., 2012; Whittaker & Fernandez‐palacios, 2007). The slopes of SARs for habitat islands are basically lower than those for true islands, which is potentially due to the fact that habitat islands are less isolated and have increased immigration rates (Matthews et al., 2016; Whittaker & Fernandez‐palacios, 2007). The slope of SARs for total bryophytes in TIL was 0.3565, which fell within the typical range for true islands, indicating that there were isolation effects of fragmented forests on bryophyte assemblages to an extent. Gould (1979) suggested comparing SARs built for different biotas in the same region to investigate how different groups respond to the same eco‐geographical condition. In TIL, the z value of SAR for bryophytes was larger than those for the other eight biotas (Table 6). In the study region, the z value for bryophytes was higher than those of the other biotas, indicating stronger effects of dispersal limitation on bryophytes than on other biotas in the study region.

### 4.5 About the index of isolation

The colonization of a given taxon on an island is a process occurring through immigration from the mainland, where the total species pool is available for colonizing new areas (MacArthur & Wilson, 1967). In fragmented landscapes, each island has its potential species pool of immigrants, and the isolation of an island depends not only on the distance to the mainland (with major species pool) but also on the amount of habitat within some distance of the given island (with local species pool, Fahrig, 2013). The amount of habitat within a given buffer around a given island was an ideal measure of the island's isolation (Bender et al., 2003; Tischendorf et al., 2003). Berglund and Jonsson (2001) used this index as a surrogate for isolation degree to study the effects of isolation on vascular plants and fungal species richness. Furthermore, in the study region, most islands were multilong branched in appearance (Figure S1). It was thus inappropriate to only take the nearest distance of a given island from the shore as a measure of its isolation degree. Therefore, according to the special landscapes in the study region, besides ISD, ISW was also included as a surrogate of isolation degree for a given island.

According to the data of the 11 major families in Tables 4 and 5, overall, the spatial effects are significantly and positively correlated with the isolation effects (*r* = .795, *n* = 11, *p* < .005). However, the isolation effects did not show significant dispersal limitations on SC for seven of 11 families (*p* > .05, Table 5). The possible reasons were due to the relatively short distance from the shore for most islands and the long‐ and multibranched appearance of many islands in the study region (Figure S1). The relatively short distance and long‐ and multibranched island appearance make both ISD and ISW improperly reflect the degree of isolation for these islands. Therefore, spatial eigenvectors are better than the isolation indices in testing the effects of dispersal limitation on community assemblage in similar land‐bridge islands.

### 4.6 About the choice of null models

To explore the mechanisms of species assembly, null models were often used to simulate expected beta diversity on the islands, where species could disperse and colonize randomly (Chase et al., 2011; Liu et al., 2020; Segre et al., 2014). However, which combination of metrics and null models should be used in each particular circumstance is a matter of debate (Ulrich et al., 2009; Ulrich & Gotelli, 2007). In this study, we used six null models to evaluate the significance of the beta diversity for the bryophytes in the TIL. The algorithms of these null models differ in whether rows and columns are treated as fixed sums, equiprobable, or proportional (Gotelli, 2000). We found that significant differences between observed and expected values were detected using the EF, FE, PE, PF, and FF null models, but no expected values of beta diversity were obtained for some families because of empty columns in the randomized matrices generated by using the EE null model. According to Gotelli (2000), the FE and FF null models that maintain fixed row sums (species in a row) were invulnerable to Type I errors (false positives). Therefore, the FE, FF, EF, PE, and PF null models, especially the former two null models could be used in the studies relevant to similar topics in the future.

## 5 CONCLUSION

Although bryophytes are considered to have a long‐distance dispersal capacity, some previous studies provided sporadic evidence that dispersal limitation was not a major driver of bryophyte assemblages. However, our multiple perspective studies in the geographical range of ca. 350 ha detected strong and significant effects of dispersal limitation on bryophyte assemblages. Dispersal limitation contributed more to SC variation than environmental filtering, not only for the bryophytes as a whole but also for other major categories and nine of the 11 major families. The contribution of dispersal limitation to SC variation was more important in liverworts than in mosses, in pleurocarpous mosses than in acrocarpous mosses, and highly varied among major families. Therefore, it was dispersal limitation rather than environmental filtering that predominantly regulated bryophyte assemblages in the fragmented subtropical forests. The effects of dispersal limitation on bryophyte assemblages varied among different taxa, namely were taxon‐dependent.

## AUTHOR CONTRIBUTIONS

**Dandan Li:** Data curation (equal); methodology (equal); writing – review and editing (equal). **Feng Zhang:** Data curation (equal); formal analysis (lead); methodology (equal); software (lead); writing – review and editing (equal). **Guangyu Luo:** Data curation (equal); formal analysis (equal); methodology (equal); software (equal); writing – original draft (equal). **Ting Zhang:** Data curation (equal); methodology (equal); software (equal). **Jinqiao Lv:** Data curation (equal); methodology (equal). **Wenchao Wang:** Data curation (equal); methodology (equal). **Jun Yang:** Data curation (equal); methodology (equal). **Dejun You:** Conceptualization (equal); data curation (equal); methodology (equal). **Nanlong Xu:** Data curation (equal); methodology (equal). **Shuiliang Guo:** Conceptualization (equal); data curation (equal); funding acquisition (lead); methodology (equal); supervision (equal); writing – original draft (lead); writing – review and editing (lead). **Jing Yu:** Conceptualization (equal); funding acquisition (equal); methodology (equal).

## CONFLICT OF INTEREST STATEMENT

The authors declare that they have no known competing financial interests or personal relationships that could have influenced the work reported in this study.

## Supporting information


Figure S1

Figure S2

Table S1

Table S2

Table S3

Table S4

Table S5

## Data Availability

All data are archived in the Dryad Digital Repository at https://doi.org/10.5061/dryad.msbcc2g2k.
