# Supplementary figures and images for: Taxon‐dependent effects of dispersal limitation versus environmental filters on bryophyte assemblages―Multiple perspective studies in land‐bridge islands
Source: Ecol Evol. 2023 Feb 24;13(2):e9844. doi: 10.1002/ece3.9844 (PMC9951200; doi:10.1002/ece3.9844)

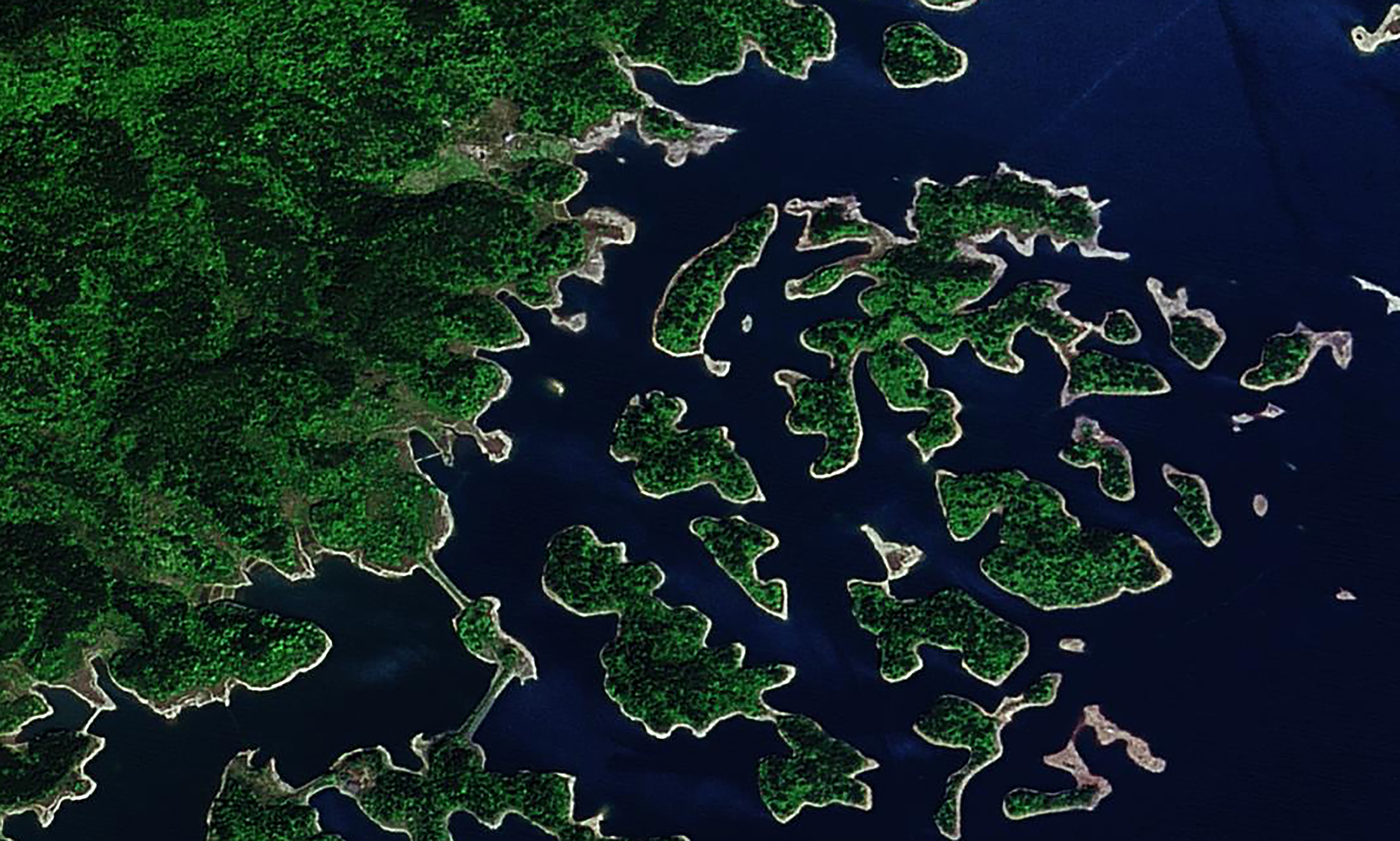

Supplement: Supplementary file 1 — Figure S1 [file ECE3-13-e9844-s002.tif]

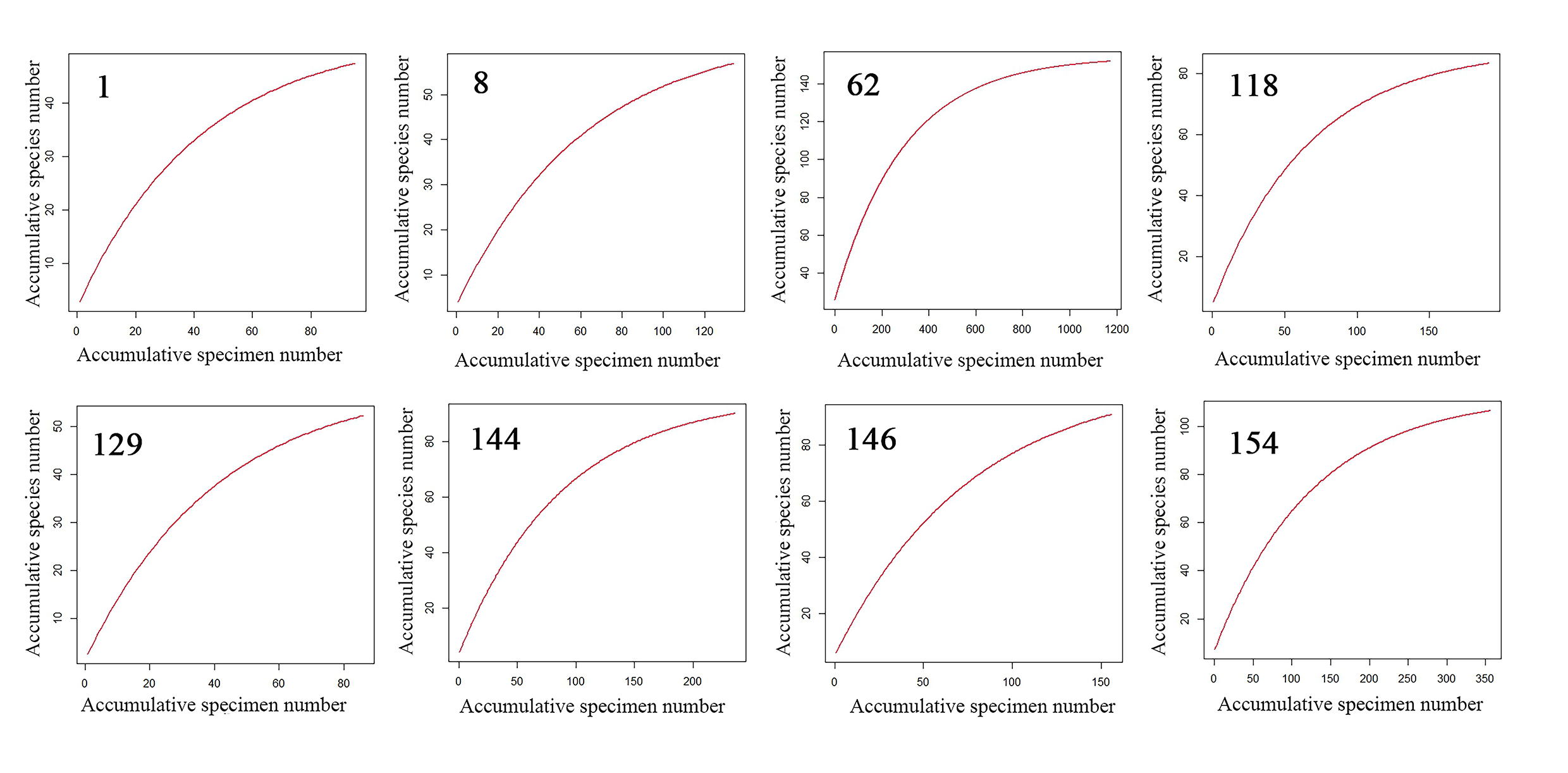

Supplement: Supplementary file 2 — Figure S2 [file ECE3-13-e9844-s003.tif]
