# Supplementary material for: Taxon‐dependent effects of dispersal limitation versus environmental filters on bryophyte assemblages―Multiple perspective studies in land‐bridge islands
Source: Ecol Evol. 2023 Feb 24;13(2):e9844. doi: 10.1002/ece3.9844 (PMC9951200; doi:10.1002/ece3.9844)
Supplement: Supplementary file 6 — Table S4 [file ECE3-13-e9844-s005.docx]

Table S4. Significance levels of differences in beta diversity among eleven major families of bryophytes (*P*-value)

| Families | ANO | BRA | BRY | ENT | FIS | HYP | LEU | LOP | MNI |
| --- | --- | --- | --- | --- | --- | --- | --- | --- | --- |
| BRA | * |  |  |  |  |  |  |  |  |
| BRY | * | * |  |  |  |  |  |  |  |
| ENT | * | * | 0.715 |  |  |  |  |  |  |
| FIS | * | * | 0.188 | 0.250 |  |  |  |  |  |
| HYP | * | * | * | * | * |  |  |  |  |
| LEU | * | * | * | * | * | * |  |  |  |
| LOP | * | * | * | * | * | * | ***** |  |  |
| MNI | * | * | * | * | * | * | * | * |  |
| POT | * | * | * | * | * | * | * | 0.519 | * |

Note: ANO = Anomodontaceae, BRA = Brachytheciaceae, BRY = Bryaceae, ENT = Entodontaceae, FIS = Fissidentaceae, HYP = Hypnaceae, LEU = Leucobryaceae, LOP = Lophocoleaceae, MNI = Mniaceae, POT = Pottiaceae; *: *P* < 0.001
