# Supplementary material for: Taxon‐dependent effects of dispersal limitation versus environmental filters on bryophyte assemblages―Multiple perspective studies in land‐bridge islands
Source: Ecol Evol. 2023 Feb 24;13(2):e9844. doi: 10.1002/ece3.9844 (PMC9951200; doi:10.1002/ece3.9844)
Supplement: Supplementary file 7 — Table S5 [file ECE3-13-e9844-s007.docx]

Table S5. Relationships of accumulative species number with accumulative sampling specimens for eight largest islands.

| Island no. /area (ha) | Number of observed species /specimen number | Asymptotic models | Expected species number | Error % |
| --- | --- | --- | --- | --- |
| 1/ 76.20 | 48/95 | Y = 52.68 – 51*exp(-exp(-3.74)*x) | 52.68 | 8.8838 |
| 8/ 137.95 | 58/134 | Y = 63.80 – 60.6*exp(-exp(-3.74)*x) | 63.8 | 9.0923 |
| 62/ 1242.39 | 158/1172 | Y = 160.22 – 134.51*exp(-exp(-5.68)*x) | 160.22 | 1.3856 |
| 118/ 97.54 | 85/191 | Y = 88.12 – 84.22*exp(-exp(-4.2)*x) | 88.12 | 3.5439 |
| 129/ 51.11 | 53/86 | Y = 59.33 – 58.09*exp(-exp(-3.7)*x) | 59.33 | 10.6616 |
| 144/ 65.26 | 92/236 | Y = 96.50 – 93.23*exp(-exp(-4.47)*x) | 96.5 | 4.6632 |
| 146/ 37.23 | 93/156 | Y = 104.10 – 99.27*exp(-exp(-4.34)*x) | 104.1 | 10.6628 |
| 154/ 869.03 | 109/356 | Y = 112.72 – 106.1*exp(-exp(-4.83)*x) | 112.72 | 3.3002 |

Sampling error = (E – O) / E * 100%, E: Maximum expected total species number, O: Observed species number.
